# Supplementary material for: The interactome of intact mitochondria by cross-linking mass spectrometry provides evidence for coexisting respiratory supercomplexes
Source: Mol Cell Proteomics. 2017 Dec 8;17(2):216–32. doi: 10.1074/mcp.RA117.000470 (PMC5795388; doi:10.1074/mcp.RA117.000470)
Supplement: Supplemental Data [file supp_RA117.000470_133922_0_supp_23978_fzffwf.zip › spectra_annotation/mito_DR_spectra_annotation/4-1-12-1-12-1.pdf]

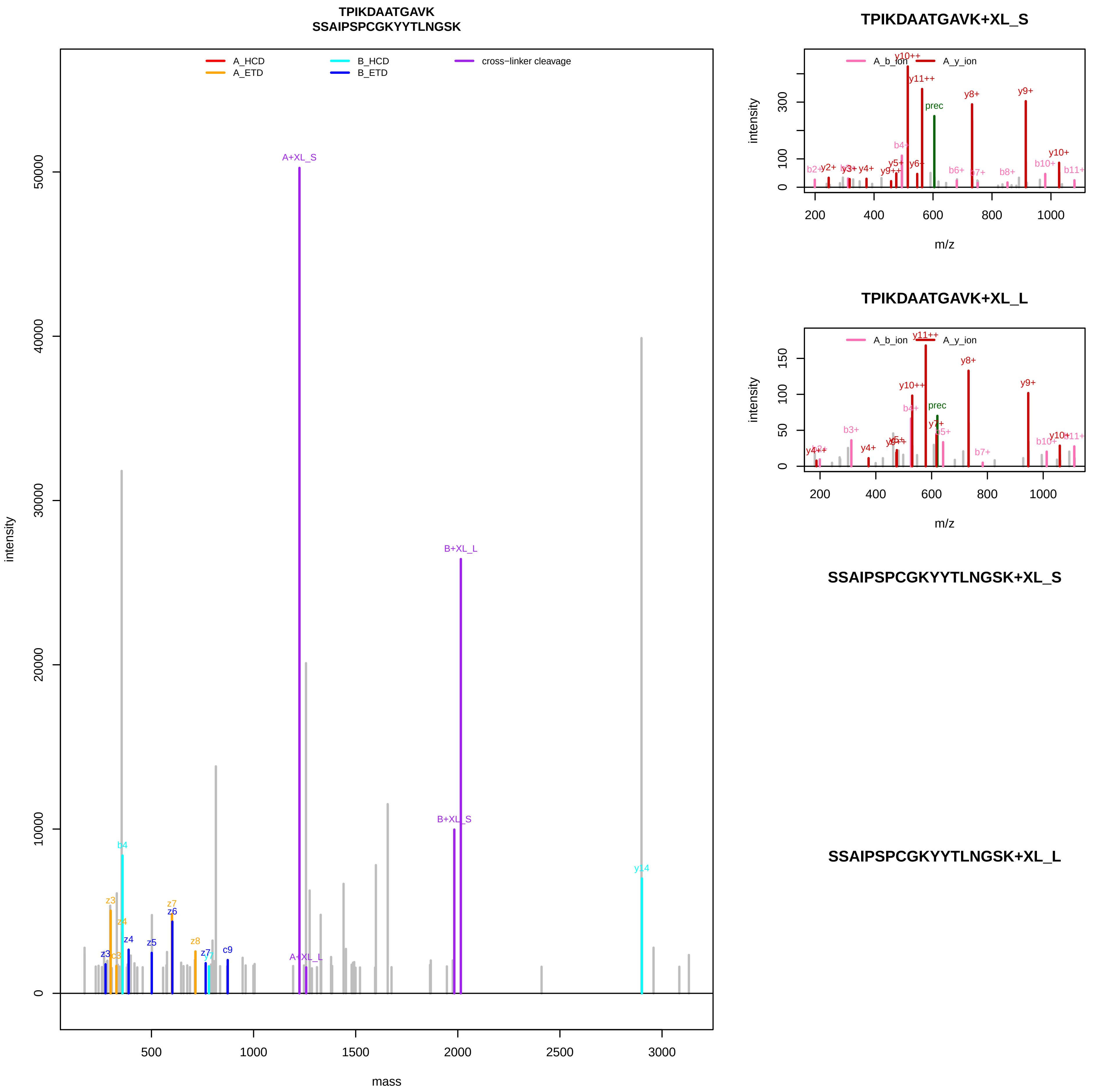
TPIKDAATGAVK
SSAIPSPCGKYYTLNGSK
A_HCD
A_ETD
B_HCD
B_ETD
cross−linker cleavage
A+XL_S
B+XL_L
B+XL_S
A+XL_L
b4
y14
z3
c3
z4
z5
z6
z7
z8
c9
y7
intensity
mass
0
10000
20000
30000
40000
50000
500
1000
1500
2000
2500
3000
TPIKDAATGAVK+XL_S
A_b_ion
A_y_ion
y10++
y11++
prec
y8+
y9+
b4+
y10+
b10+
b11+
b2+
y2+
y3+
y4+
y5+
y6+
y9++
b6+
b7+
b8+
intensity
m/z
0
100
300
200
400
600
800
1000
TPIKDAATGAVK+XL_L
A_b_ion
A_y_ion
y11++
y8+
y10++
y9+
prec
b4+
b3+
y7+
b5+
y4+
y4++
b2+
b7+
b10+
y10+
b11+
intensity
m/z
0
50
100
150
200
400
600
800
1000
SSAIPSPCGKYYTLNGSK+XL_S
SSAIPSPCGKYYTLNGSK+XL_L
